# Anti-GBM Nephritis in an 11-Year-Old Female Child: A Rare Case Report

**DOI:** 10.7759/cureus.67736

**Published:** 2024-08-25

**Authors:** Shivani Kale, Manojkumar Patil, Shailaja Mane, Neha Thorbole, Manoj Matnani

**Affiliations:** 1 Paediatrics, Dr. D. Y. Patil Medical College, Hospital and Research Centre, Pune, IND

**Keywords:** autoantibodies, autoimmune, goodpasture's syndrome, nephritis, anti-glomerular basement membrane disease

## Abstract

Anti-glomerular basement membrane (GBM) nephritis is a rare autoimmune condition involving the glomerular basement membrane of the kidneys. This case report describes an 11-year-old female who presented with edema, decreased urine output, and altered sensorium, progressing to hypertension and requiring emergent hemodialysis. A renal biopsy showing Immunoglobulin G (IgG) linear deposits confirmed the diagnosis. The patient was treated with intravenous methylprednisolone and antihypertensives and then scheduled for regular dialysis. This case underscores the critical need for early diagnosis and aggressive management to prevent severe complications in pediatric anti-GBM disease.

## Introduction

Anti-glomerular basement membrane (GBM) nephritis is a small-vessel vasculitis disease characterized by developing autoantibodies against the GBM of the kidneys [1,2]. This condition typically presents as rapidly progressive glomerulonephritis (RPGN) with symptoms such as hematuria, proteinuria, and renal impairment [3]. A genetic association has been reported between Human Leukocyte Antigens (HLA) *DRB11051 *and *DRB11502 *and the disease, while *HLA-DR7* and *DR1 *appear to provide some protective effect [4]. Diagnosis involves detecting either serum anti-GBM antibodies or through histological examination [5]. Treatment in pediatric patients is based majorly on limited data from adult cases. Generally, it includes acute apheresis to quickly remove the circulating factors, combined with immune-suppressor medication such as intravenous corticosteroids and cyclophosphamide [6]. Additionally, there is increasing interest in using biological agents for B cell depletion. However, the evidence supporting these treatments in pediatric patients remains very limited [5]. Goodpasture syndrome, also known as anti-GBM disease, is a rare autoimmune disorder characterized by the production of autoantibodies targeting antigens in the glomerular and alveolar basement membranes [1]. Goodpasture syndrome, or anti-GBM disease, remains a very rare disease entity in the pediatric population characterized by the presence of pulmonary hemorrhage and rapidly evolving glomerulonephritis [7].

## Case presentation

An 11-year-old female (with weight 46 kg and height 156 cm), born as the second child from a non-consanguineous marriage, presented with a history of decreased urine output for 12 days, periorbital and pedal edema for 10 days, and altered sensorium for the past 1-2 days. Initially, the child developed pedal edema and a marked decrease in urine output 12 days prior, with a recent onset of altered sensorium. It prompted a visit to a local hospital where she was seen by an adult nephrologist. The nephrologist advised the child to undergo a renal biopsy. However, the family refused treatment and discharged the child against medical advice. Subsequently referred to our hospital, the patient was admitted to the pediatric intensive care unit (PICU).

**Figure 1:**
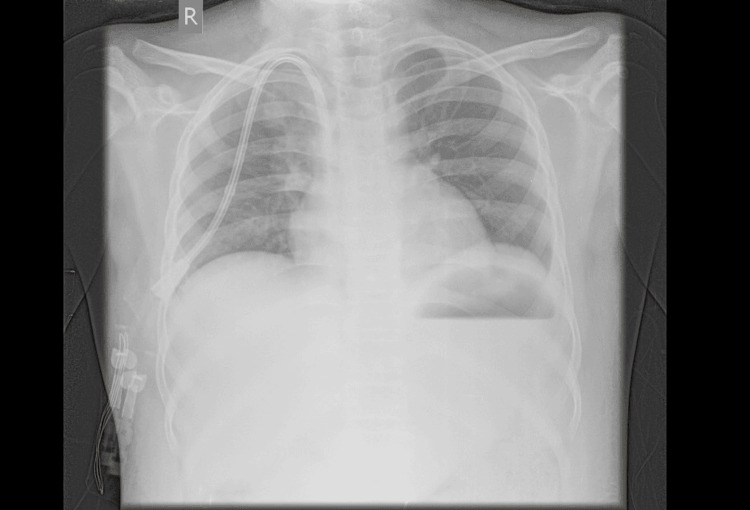
PA view of chest radiograph PA: Posteroanterior

Upon examination, the patient exhibited a pulse rate of 71 beats per minute, hypertensive readings of 149/92 mmHg (>99 percentile), respiratory rate of 20 breaths per minute, and an oxygen saturation of 98% on room air. The Glasgow Coma Scale (GCS) score was 12 (E4V4M4). The patient had complaints of blurring vision along with hypertensive readings. Fundoscopic examination revealed no papilledema. Auscultation revealed bilaterally equal breath sounds with no cardiac murmurs (Figure 1). On abdominal palpation, the abdomen was soft, non-tender, and without organomegaly. There was bilateral ankle pitting edema observed. There was no reported history of shortness of breath, hemoptysis, or chest pain. There was no history of any preceding infection.

Urine analysis demonstrated significant findings: urine albumin was markedly positive (++++) with substantial proteinuria indicated by a 24-hour urinary protein level of 3173 mg (normal = <150 mg) and an elevated urine protein-to-creatinine ratio of 1.5625 (normal = <0.5), indicating a marked loss of protein in the urine. Hematuria was present with 8-10 red blood cells per high power field (hpf), while the white blood cell count was normal at 2-3/hpf. Immunological tests showed antineutrophil cytoplasmic antibodies - cytoplasmic pattern (ANCA-C) levels of <3.0 (normal <12) and antineutrophil cytoplasmic antibodies - perinuclear pattern (ANCA-P) levels of 3.1 (normal = <12). The antistreptolysin O (ASO) titer was <50.0 (normal = 0-200) (Table 1).

**Table 1:** Immunological test results ANCA-C: Antineutrophil cytoplasmic antibodies (cytoplasmic pattern); ANCA-P: Antineutrophil cytoplasmic antibodies (perinuclear pattern); ASO: Antistreptolysin O

Parameter	Result	Reference Range
ANCA-C	<3.0	<12
ANCA-P	3.1	<12
ASO titer	<50.0	0–200

**Figure 2:**
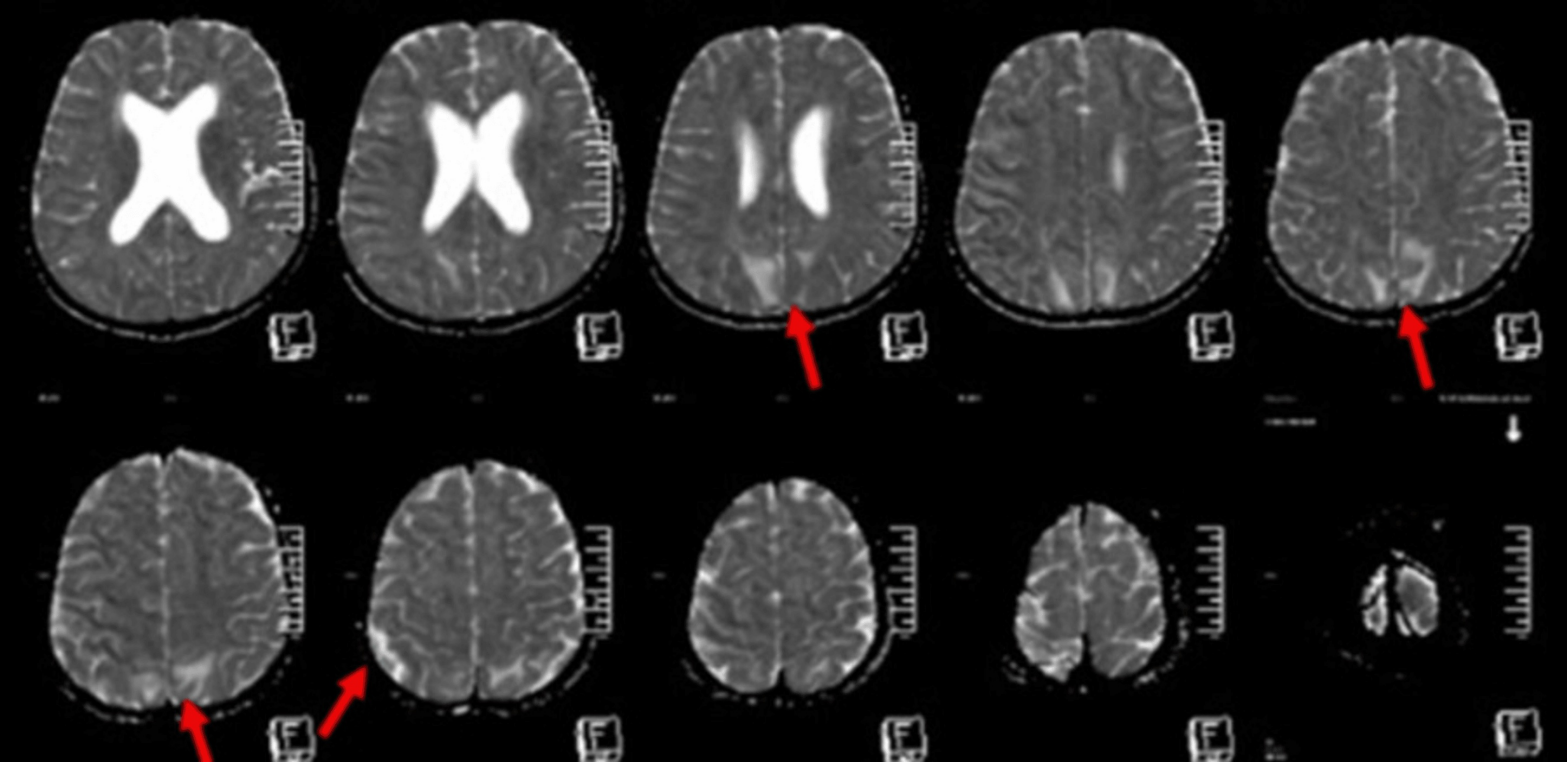
Brain MRI. Axial section showing diffuse vasogenic edema noted in the subcortical and deep white matter of bilateral parieto-occipital lobes (red arrow). MRI: Magnetic resonance imaging

The patient’s MRI suggested posterior reversible encephalopathy syndrome (PRES), characterized by diffuse vasogenic edema in the subcortical and deep white matter of the bilateral parieto-occipital lobes, likely secondary to severe hypertension (Figure 2). The hypertension was effectively managed with a combination of three antihypertensives: amlodipine, labetalol, and prazosin.

**Figure 3:**
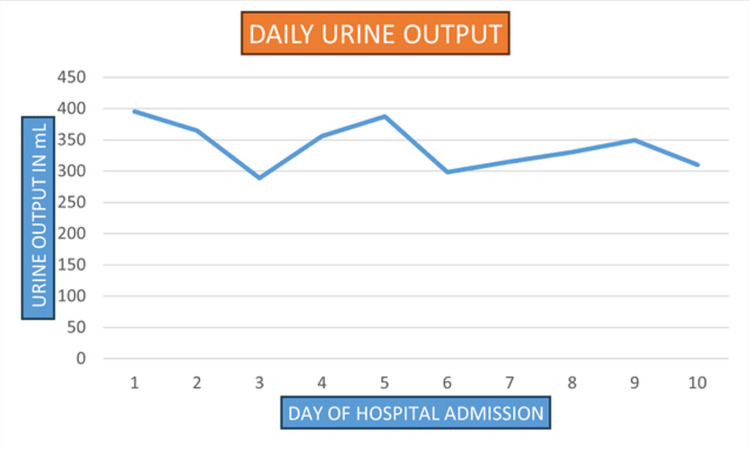
Trend of daily urine output during admission

**Figure 4:**
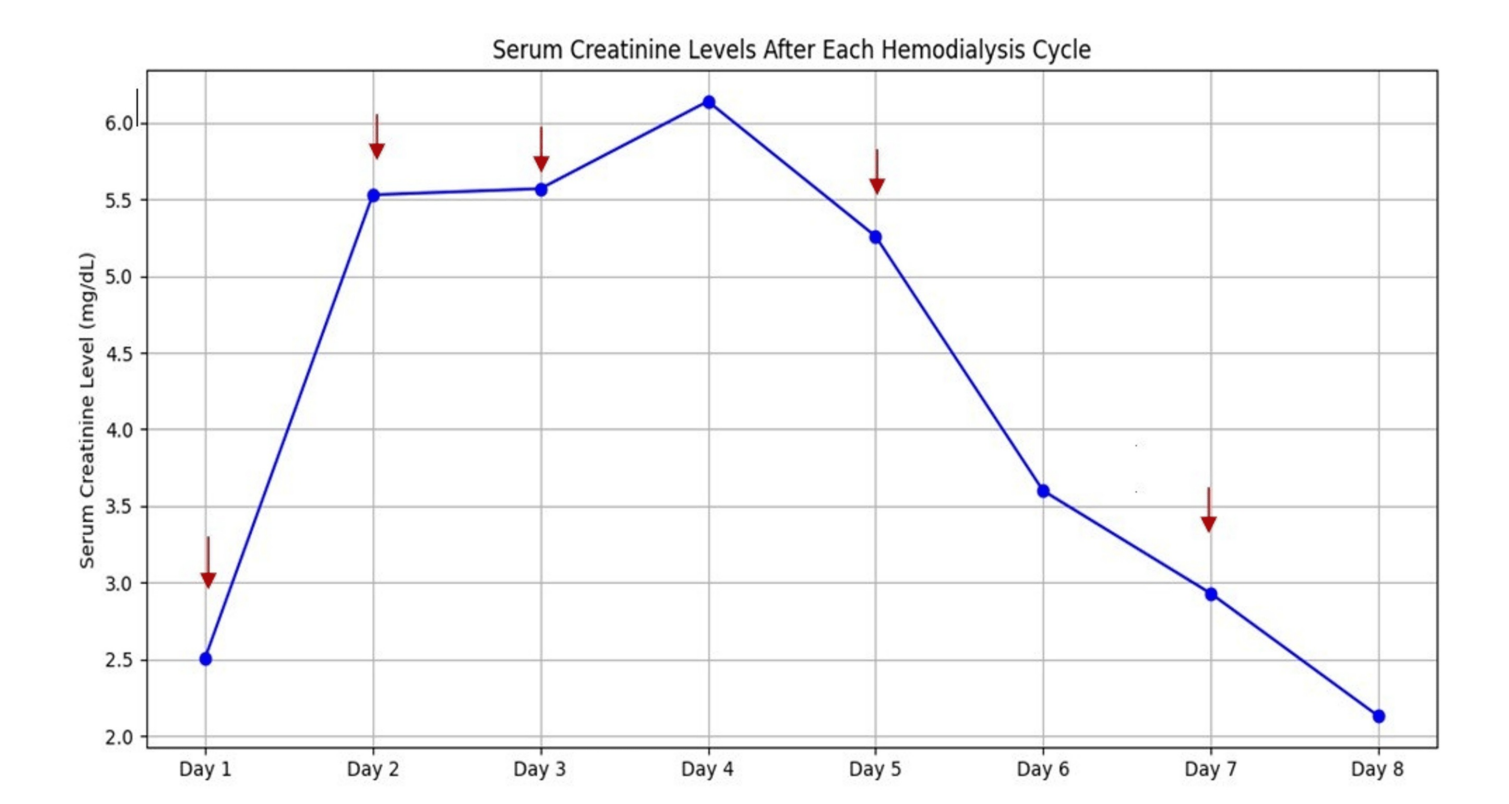
Serum creatinine level with each hemodialysis cycle. Arrows indicate hemodialysis cycles.

The hematology results revealed an abnormal hemoglobin level (7.1 g/dL) along with a hematocrit of 23%. The total leukocyte count was 15,200/µL with a high neutrophil-to-lymphocyte ratio (86/8) (Table 2). The platelet count was 21,500 /µL. There was a serial rise in serum creatinine with continued low urine output for which the patient underwent emergent hemodialysis (Figures 3-4). The patient received one packed cell volume (PCV) transfusion during a cycle of hemodialysis.

**Table 2:** Laboratory investigations

	Result	Reference Range
Hemoglobin	7.10	12.0–14.5 g/dL
Hematocrit	23%	35.7–43.0%
White blood cells	15,200 total (differential count: neutrophils 86%, lymphocytes 8%)	4000–10,800/uL
Platelet count	215,000	150,000–410,000/uL
Sodium	141	135 to 145 mEq/L
Potassium	4.5	3.5 to 5.0 mEq/L
Chloride	111	96 to 106 mEq/L
Blood urea nitrogen	235	17–49 mg/dL
Total protein	4.6	6.0 to 8.3 g/dL
Albumin	2.80	3.5 to 5.0 g/dL
Globulin	1.8	2.0 to 3.5 g/dL
Calcium	8.80	8.5 to 10.2 mg/dL
Magnesium	2.40	1.7 to 2.2 mg/dL
Venous Blood Gas		
pH	7.42	7.32–7.42
pCO_2_	25	41–51 mmhg
pO_2_	56	25–40 mmhg
Lactate	0.4	<1 mmol/L
Bicarbonate	20.6	21–28 mEq/L
SO_2_	90%	

**Figure 5:**
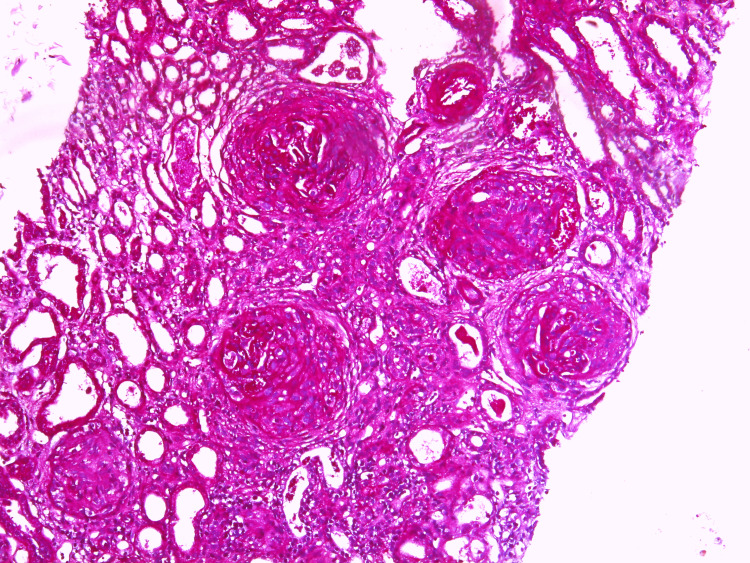
PAS-stained renal biopsy (100x) image showing fibrous crescents PAS: Periodic acid-Schiff

**Figure 6:**
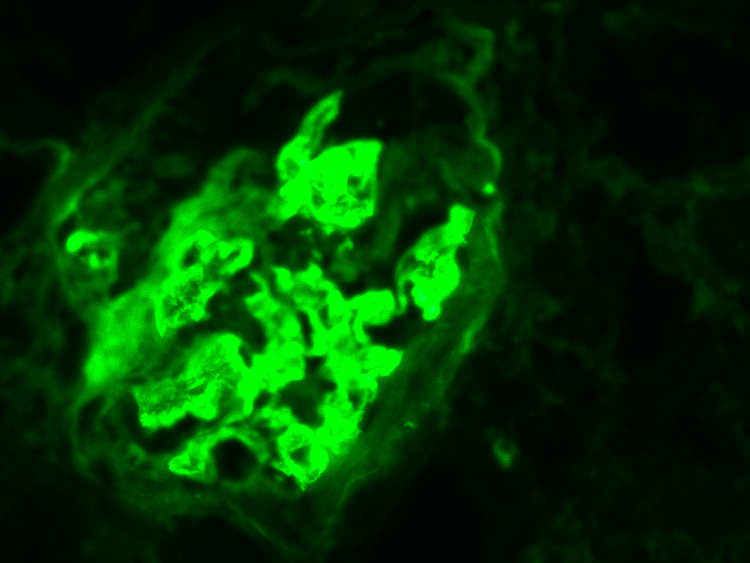
Immunofluorescence showing IgG positive (+++) linear deposits along the glomerular capillary wall. IgA, IgM, C3, C1q are negative.

The renal biopsy revealed significant chronic damage. Of the 17 glomeruli examined, 13 showed global sclerosis and fibrous crescents (Figure 5). Interstitial fibrosis and tubular atrophy affecting 60% of the renal tissue were noted. Immunofluorescence staining showed IgG-positive (+++) linear deposits along the glomerular capillary walls (Figure 6). These findings were consistent with anti-GBM disease that had advanced to chronic glomerulonephritis.

Anti-GBM antibodies were tested to check for circulating antibodies in the patient, but the results were negative (12.6, with a normal range of 0-21.0). The test was repeated to ensure accuracy and rule out potential false negatives or laboratory errors. It yielded another negative result.

The patient was treated with intravenous albumin infusions for edema management and received pulse IV methylprednisolone (500 mg for three doses over 3 days), followed by oral steroids. Due to the chronicity of the condition and the patient's poor socio-economic status, plasmapheresis was not initiated. Antihypertensive treatment was started with amlodipine 10 mg daily, labetalol 100 mg twice daily, and prazosin 5 mg twice daily. The patient continued to be oliguric, with urine output less than 400 ml (Figure 3). The patient was prepared for discharge after securing a perm-catheter for regular hemodialysis. Upon discharge, the patient continued on oral prednisolone 40 mg daily, with amlodipine 10 mg daily, labetalol 100 mg two times daily, and prazosin 5 mg two times daily.

## Discussion

Anti-GBM nephritis is rare in children, yet it accounts for 20% of all RPGN cases. Determining its exact incidence in pediatric populations remains challenging. In adults, the annual incidence ranges from 0.5-1.0 cases per million individuals [2]. The disease's prevalence varies seasonally and geographically, potentially triggered by infections like influenza A. Reports during the COVID-19 pandemic have also attributed SARS-CoV-2 to the onset of this disease [8].

In 80-90% of cases, the typical presentation of the disease includes RPGN, often necessitating urgent renal replacement therapy. Renal manifestations range from hematuria and proteinuria to rapidly advancing renal failure, characterized by oliguria, fluid overload, and hypertension. This case featured initial symptoms of pedal edema, decreased urine output, altered sensorium, and hypertension. Anti-GBM, previously known as Goodpasture syndrome, is an extremely rare cause of RPGN and chronic kidney disease stage 5 (CKD5) in children [9]. Approximately 60% of patients with Goodpasture syndrome may also experience pulmonary hemorrhage, while a smaller percentage solely exhibit pulmonary involvement. Common pulmonary symptoms include shortness of breath, wheezing, coughing blood, and chest pain [10]. However, the patient did not manifest any signs or symptoms of pulmonary involvement [5]. Goodpasture syndrome is uncommon in children, with only 30 cases documented in pediatric literature, the youngest being 11 months old.

Diagnosis is done mainly by detecting anti-GBM antibodies in serum or histologically. In this case, the diagnosis was made by the presence of anti-GBM antibodies histologically by performing a renal biopsy. Currently, enzyme-based immunoassays or bead-based fluorescence assays detect circulating anti-GBM antibodies, but about 10% of patients may not show detectable antibodies. Thus, serologic testing should not be the sole diagnostic method when a kidney biopsy is feasible [4].

A similar presentation was reported by Tamura et al. where a 43-year-old Japanese woman was admitted to a hospital complaining of hematuria that had persisted for more than one month. Serological examination revealed negativity for anti-nuclear, anti-neutrophilic cytoplasmic, and anti-GBM antibodies. However, renal biopsy showed cellular crescents. Immunofluorescence revealed strong diffuse linear capillary loop staining for IgG. Accurately identifying the presence of anti-GBM disease is important to initiate optimal treatment [10].

The cases of dual positivity for ANCA and anti-GBM antibodies are reported frequently. A retrospective review by Bassam et al. had 43 patients diagnosed with the anti-GBM disease over 20 years in two centers, including nine with dual anti-GBM and ANCA positivity. Renal biopsies from 27 patients were scored for the presence of active and chronic lesions. Dual-positive patients were almost 20 years older than those with anti-GBM positivity alone (P = 0.003). The overall 1-year patient and renal survivals were 88 and 16%, respectively [11]. It was proposed that the initial presence of ANCA may expose the alpha-3 chain epitope [12]. This condition is linked to a poorer prognosis, necessitating early aggressive treatment [13]. In this case, ANCA was negative, ruling it out as a differential diagnosis.

Management principles encompass several strategies such as removing harmful circulating antibodies and immune mediators via acute apheresis; inhibiting antibody production with B cell-depleting agents and/or cyclophosphamide; and reducing inflammation using corticosteroids and immune-modifying agents [14]. The treatment of anti-GBM disease in pediatric patients mirrors the interventions used for adults, as recommended in the recent Kidney Disease Improving Global Outcomes (KDIGO) glomerular disease guidelines [15].

In this case, the patient was initially treated with pulse IV methylprednisolone at 500 mg for three doses over three days, followed by oral steroids. Considering the chronic nature of the condition and the patient's poor socio-economic status, plasmapheresis was not pursued.

## Conclusions

This case highlights the complexities and challenges in managing a pediatric patient with advanced anti-GBM disease exacerbated by delayed appropriate treatment. The 11-year-old female presented with severe renal failure, hypertension, and altered sensorium with blurring of vision secondary to PRES. Despite the advanced stage of her condition and socio-economic constraints, the patient was stabilized with intravenous methylprednisolone, oral steroids, and a combination of antihypertensive medications. Intensive immunosuppression and plasmapheresis were not feasible due to the chronicity of the disease and socio-economic factors. The patient's condition necessitated regular hemodialysis, and she was discharged with a permcath in place for ongoing dialysis, alongside a regimen of oral steroids and antihypertensives. The chronic nature of the patient's condition, coupled with the presence of significant renal damage, underscores the importance of early intervention and adherence to evidence-based medical treatment to prevent irreversible outcomes in similar cases.
